# Structure of Human Complement C8, a Precursor to Membrane Attack

**DOI:** 10.1016/j.jmb.2010.10.031

**Published:** 2011-01-14

**Authors:** Doryen Bubeck, Pietro Roversi, Rossen Donev, B. Paul Morgan, Oscar Llorca, Susan M. Lea

**Affiliations:** 1 Division of Structural Biology, University of Oxford, Wellcome Trust Centre for Human Genetics, Roosevelt Drive, Oxford OX3 7BN, UK; 2 Sir William Dunn School of Pathology, University of Oxford, South Parks Road, Oxford OX1 3RE, UK; 3 Department of Medical Biochemistry and Immunology, School of Medicine, Cardiff University, Heath Park, Cardiff CF14 4XN, UK; 4 Centro de Investigaciones Biológicas (CIB), Spanish National Research Council (CSIC), Ramiro de Maeztu, 9. 28040 Madrid, Spain

**Keywords:** MAC, membrane attack complex, MACPF, membrane attack complex/perforin, RCT, random conical tilt, 2D, two-dimensional, C8, complement, 3D electron microscopy, terminal pathway, membrane attack complex (MAC)

## Abstract

Complement component C8 plays a pivotal role in the formation of the membrane attack complex (MAC), an important antibacterial immune effector. C8 initiates membrane penetration and coordinates MAC pore formation. High-resolution structures of C8 subunits have provided some insight into the function of the C8 heterotrimer; however, there is no structural information describing how the intersubunit organization facilitates MAC assembly. We have determined the structure of C8 by electron microscopy and fitted the C8α-MACPF (membrane attack complex/perforin)-C8γ co-crystal structure and a homology model for C8β-MACPF into the density. Here, we demonstrate that both the C8γ protrusion and the C8α-MACPF region that inserts into the membrane upon activation are accessible.

## Graphical Abstract

**Figure:**
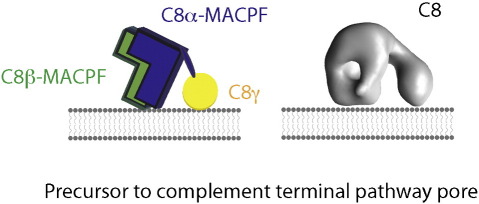

**Research Highlights:**

► Complement component C8 initiates membrane penetration of the membrane attack complex, an important innate immune effector. ► The structure of C8 reveals a core domain with a globular protrusion. ► Docking of pseudo-atomic models into the electron microscopy reconstruction indicates that C8γ projects away from the C8αβ core, in which the predicted transmembrane residues of C8α-MACPF and one face of the C8β-MACPF are surface exposed.

The membrane attack complex (MAC) is a macromolecular pore that targets and lyses pathogens that challenge the host. Deposition of these pores on human cells contributes to tissue damage in a variety of diseases such as multiple sclerosis.^1,2^ C8 plays a central role in MAC assembly by coordinating the interaction with MAC precursor C5b-7 and the pore-forming protein C9. It is also the first component to penetrate the lipid bilayer.

C8 is a 150-kDa complex composed of three subunits: α (64 kDa), β (64 kDa), and γ (22 kDa). C8α and C8β are highly homologous and, like several other MAC components, contain a common membrane attack complex/perforin (MACPF) domain. The crystal structure of C8α-MACPF^3,4^ is characterized by an L-shaped fold in which two subregions, TMH1 and TMH2, are similar to those in cholesterol-dependent cytolysins that undergo a conformational change to insert into membranes.^3^ C8γ, on the other hand, has a lipocalin fold and shares no homology with any other complement protein.^5^ The C8α-MACPF domain coordinates the interaction with C8γ and C8β in the complex. The β-hairpin extension from the C8α-MACPF (absent in C8β) is disulfide bonded to C8γ.^5^ The interaction of C8α and C8β is less well characterized; however, binding assays using purified recombinant proteins define the interaction between C8β and the C8α-γ heterodimer to be primarily mediated by the two MACPF domains.^6^

C8α and C8β have unique and essential roles in the formation of hemolytic MAC. The MACPF domain of C8β is responsible for integrating C8 with the pre-assembled MAC precursor, C5b-7,^7^ while the MACPF domain of C8α undergoes a conformational change to insert into membranes and is the principal binding site for C9, the pore-forming component of the MAC.^6^ CD59, the sole membrane-bound regulator of MAC assembly, blocks the C8–C9 interaction, subsequently inhibiting C9 oligomerization and MAC-mediated lysis.^8,9^ To understand how the arrangement of C8 subunits influences the assembly of the MAC, we set out to solve the structure of C8 by three-dimensional (3D) negative-stain electron microscopy.

### C8 is a compact globular assembly

C8, isolated from plasma and purified under physiological conditions,^10^ is a stable, homogenous complex composed of three subunits, C8α, C8β, and C8γ, in which the intersubunit interactions remain intact (Fig. S1). Negatively stained C8 complexes were visualized by electron microscopy. Raw images (Fig. 1a) were aligned using reference-free alignment and classified into groups (Fig. 1b). Two-dimensional (2D) averages show that C8 has two distinct regions. The larger of the two appears globular and pseudo-2-fold symmetric with less density in the middle. The smaller one protrudes from this core.

*Ab initio* structure determination of C8 was performed using the random conical tilt (RCT) method,^13^ as well as starting from a Gaussian blob of dimensions similar to the 2D class averages (Fig. S2). These initial models served as references for an independent and parallel refinement strategy in which the resulting reconstructions showed similar density features (Fig. S3). Handedness was determined using the RCT method and applied to all reconstructions. To reduce the potential for bias arising from a noisy RCT starting model, we initiated our refinement with a Gaussian blob. This refinement of C8 converged on a reconstruction whose resolution was 24 Å, determined by the Fourier shell correlation cutoff of 0.5 (Fig. S4). As observed in both the raw images and reference-free aligned class averages, the 3D reconstruction of C8 consists of a core domain with a globular protrusion (Fig. 2a). Furthermore, these features are apparent in the 2D projections of the reconstruction as well as averages of aligned particles corresponding to those projections (Fig. 2b). The protrusion has a diameter of 40 Å, consistent with the dimensions of the C8γ lipocalin.^5^ Each face of the larger domain has a length and width of approximately 75 and 50 Å, respectively. These dimensions are compatible with the signature L-shaped domain associated with the MACPF fold.^3,4,15^

### Intersubunit organization of C8

The C8α-MACPF-C8γ heterodimer is a rigid complex, covalently linked through a disulfide bond.^4^ Assignment of C8γ to the protrusion limits the possible orientations of the C8α-MACPF-C8γ complex in the reconstruction and gives an unambiguous docking (Fig. 3; Table S1). Because of the resolution of the reconstruction, we have scored two alternate placements of C8β-MACPF in the remaining density, refinement residuals of 40% and 38%, respectively (Fig. S5 and Table S1). Both solutions are plausible, given the previous biochemical data characterizing the interactions between C8 subunits.^6^ However, only one of these solutions, which corresponds to the lower refinement residual, is consistent with a solvent-accessible glycosylation site predicted for C8β-MACPF (NetNGlyc 1.0 Server). In all models, C8γ protrudes from the core of C8, and the two MACPF domains are sandwiched together. As a result, C8γ and the membrane inserting residues of C8α-MACPF do not form an interface with C8β-MACPF (Fig. 3).

In C8α and C8β, the central MACPF domain is flanked at the N terminus by a thrombospondin type 1 domain and a class A low-density lipoprotein receptor domain, and at the C terminus by an epidermal growth factor domain and another thrombospondin type 1 domain^19^ (Fig. S6). These additional domains are thought to have a role in coordinating the complex as well as mediating hemolytic activity.^20^ Given the small size of the domains and the resolution of the reconstruction, we have not included them in our refinement. However, our map contains additional density extending from the N and C termini of both MACPF domains that is unoccupied by our current model and could correspond to these modules (Fig. 3).

The complement response culminates in the stepwise assembly of the multi-protein MAC. The MACPF domains of both C8α and C8β are required for lysis; however, membrane insertion has been attributed only to C8α-MACPF. Structural homology with bacterial cytolysins^3,21^ has more explicitly identified two regions in C8α-MAPCF (TMH1 and TMH2) that are predicted to undergo a conformational change and insert into the bilayer. In this structure, we define the orientation of the C8α-MACPF-C8γ complex and demonstrate that both TMH1 and TMH2 are surface exposed. The C8β-MACPF is presented on the opposite face for assembly into the larger C5b-7 complex, while C8γ projects away from the C8αβ core.

### Accession number

Electron microscopy map has been deposited to EBI with accession no. EMD-1805.

## Figures and Tables

**Fig. 1:**
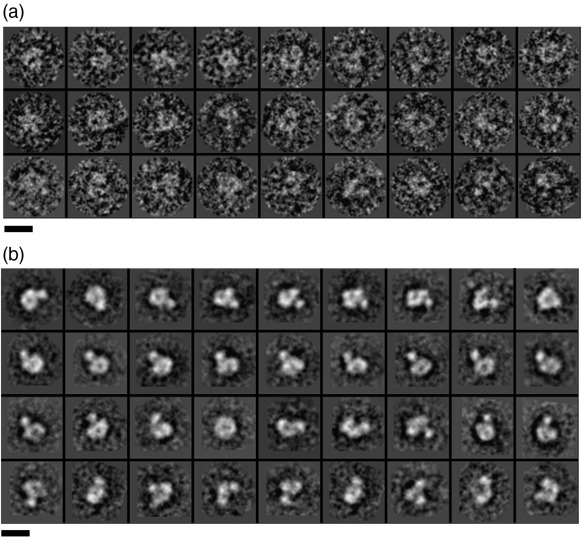
Two-dimensional images of negatively stained C8. C8 (2.5 μl of 0.03 mg/ml) was applied to a carbon-coated copper-palladium grid, glow-discharged for 10 s at 20 mA. Grids were negatively stained with 0.75% uranyl formate using the two-drop method.^11^ Images were taken under low-dose conditions (∼10 e^−^/Å^2^ per exposure) at a magnification of 59,000× on a Tecnai F30 microscope. Micrographs were digitized using a SCAI scanner (Z/I Imaging) at a step size of 7 μm and binned by a factor of 4, resulting in a pixel size of 4.74 Å/pixel (a). (b) 5167 windowed particles were subjected to 10 cycles of reference-free alignment using EMAN^12^ and classified into 362 classes. Representative 2D class averages indicate a wide range of orientations. The scale bar represents 110 Å.

**Fig. 2:**
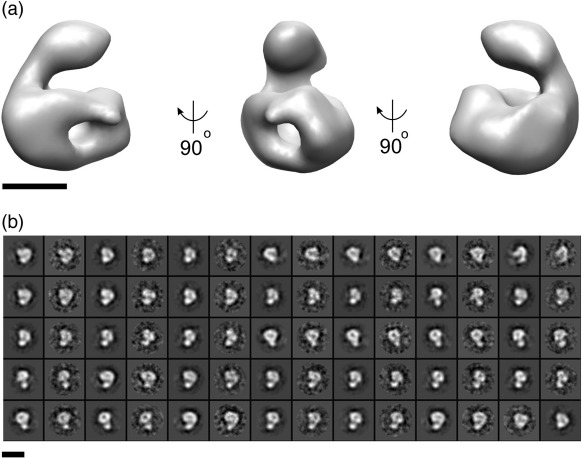
Three-dimensional reconstruction of C8 at  24-Å resolution. A Gaussian blob was used as reference to initiate the angular assignment of raw images using EMAN.^12^ The refinement was iterated until convergence, and a surface rendering of the final reconstruction is visualized from three views differing by a 90° rotation (a). Angles and axes of rotation are indicated by arrows. C8 can be described as having a pseudo-2-fold symmetric globular domain flanked by a protrusion. The scale bar represents 45 Å. Maps were visualized using CHIMERA.^14^ (b) Two-dimensional projections of the reconstruction and corresponding class averages are depicted in alternating panels, starting with the projection. The scale bar represents 110 Å.

**Fig. 3:**
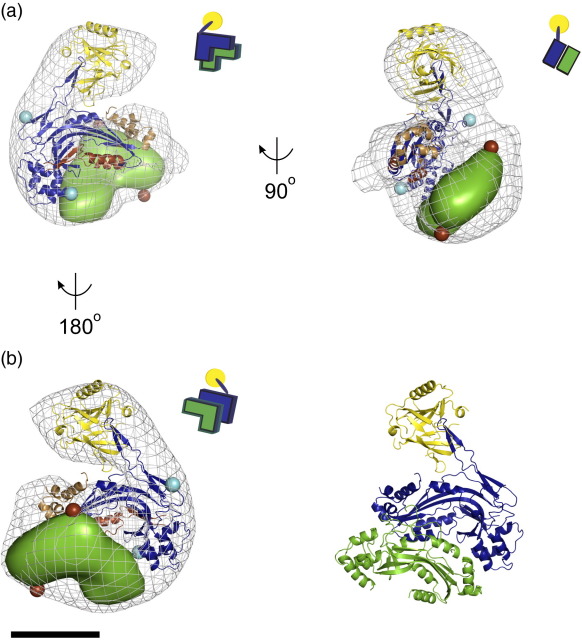
Pseudo-atomic modeling of C8. (a) The crystal structure^4^ (PDB ID: 2RD7) of the complex between C8α-MACPF (blue ribbons) and C8γ (yellow ribbons) was fit into the reconstruction (grey mesh). Using the C8α-MACPF, a homology model for C8β-MACPF (45% sequence similarity) was built with MODELLER^16^; however, loops larger than 5 residues extending from the core domain were removed. Two possible placements of C8β-MACPF, related by a 180° rotation interchanging the arms of the “L,” were scored. The one shown here corresponds to the lower refinement residual and is rendered as an isosurface filtered to 25 Å (green). Positions were refined using Chimera^14^ and PHENIX^17^ as two movable rigid bodies (see Table S1 for details). Refined models were scored against the electron microscopy map using a real space correlation coefficient computed using the BSOFT package. Access to the putative transmembrane hairpin of C8α, TMH1 (brown), and the CD59 binding site located in the TMH2 region (tan) is located on the outer face of C8. The figure was prepared using PYMOL.^18^ The N and C termini of C8α-MACPF are cyan spheres; the N and C termini of C8β-MACPF are brown spheres. Quaternary structure schematics are shown for each view. Domains are color coded similarly. The scale bar represents 45 Å. (b) Cartoon representation of the model color coded as in panel (a).
