# Supplementary material for: Structure of Human Complement C8, a Precursor to Membrane Attack
Source: J Mol Biol. 2011 Jan 14;405(2-2):325–30. doi: 10.1016/j.jmb.2010.10.031 (PMC3021121; doi:10.1016/j.jmb.2010.10.031)
Supplement: Supplementary file 1 — Supplementary Materials [file mmc1.pdf]

Supplementary information

Structure of Human Complement C8, a Precursor to Membrane Attack

Doryen Bubeck[1], Pietro Roversi[2], Rossen Donev[3], B. Paul Morgan[3‡], Oscar Llorca[4‡],

Susan M. Lea[2‡]

[1]Division of Structural Biology, University of Oxford, Wellcome Trust Centre for Human Genetics, Roosevelt Drive, Oxford OX3 7BN, UK;

[2]Sir William Dunn School of Pathology, University of Oxford, South Parks Road, Oxford OX1 3RE, UK;

[3]Department of Medical Biochemistry and Immunology, School of Medicine, Cardiff University, Heath Park, Cardiff CF14 4XN, UK;

[4]Centro de Investigaciones Biológicas (CIB), Spanish National Research Council (CSIC), Ramiro de Maeztu, 9. 28040 Madrid, Spain

‡To whom correspondence should be addressed. E-mail: susan.lea@path.ox.ac.uk, tel. 0044-1865-257385, fax 0044-1865-27556; ollorca@cib.csic.es, tel. 0091-8373112 ext 4446; morganpb@cardiff.ac.uk, tel. 0044-2920-742020

Running title: Structure of C8

Keywords: C8, complement, 3D electron microscopy, terminal pathway, membrane attack complex (MAC)

Supplementary figure legends

Fig S1. Purification of C8. Human plasma was clarified of less soluble proteins using a PEG precipitation. C8 was then isolated from the remaining solution using monoclonal anti-C8 immuno-affinity columns as described previously[1] and further purified by gel filtration on a S200 26/60 (GE Healthcare) size exclusion column in 100 mM NaCl, 0.15 mM $CaCl_2$, 0.5 mM $MgCl_2$, 5 mM Imidazole pH 7.4 (a) An FPLC chromatogram. C8 elutes as a single, monodisperse, 150kD complex on a S200 26/60 (GE Healthcare) size exclusion column. The void and bed volumes of the column are indicated. The 158 kDa and 78 kDa markers are aldolase and conalbumin, respectively (GE Healthcare Gel Filtration HMW Calibration Kit). (b) Coomassie-stained SDS NuPAGE gel (4-12% Bis-Tris) of the peak fractions. Samples run under nonreducing (lane 2) and reducing (lane 3) conditions show that purified C8 contains all three subunits and that C8a and C8? form a disulfide-bonded hetero-dimer. The calculated molecular weights of C8a, C8β, and C8? are 64 kDa, 64 kDa, and 22 kDa respectively. SeeBlue Plus2 molecular weight markers (Invitrogen) are shown in lane 1. The gel was run in a tricine buffer.

Fig S2. Initial models for refinement. A Gaussian blob with the approximate dimensions measured from the reference-free averages of C8 (a; grey) was generated using EMAN[2]. Other starting models (a; cyan and yellow) were calculated experimentally using the Random Conical Tilt (RCT) method[3]. Eighteen tilt pairs (0 and 50°) were taken at a magnification of 59,000 on a Tecnai F30 microscope, as described in Fig. 1. The 50° image was recorded first, followed by the 0° image. 1409 windowed tilt pairs were selected interactively in WEB[4], CTF-corrected using the phase-flip option in BSOFT[5],

and subjected to 5 rounds of reference-free alignment[6] before converging on a stable global average. Using hierarchical classification techniques in SPIDER[4], 0° images were separated into 100 groups generating 2D class averages. 3D reconstructions were computed from the 50° images corresponding to class averages using the back-projection method in SPIDER[4]. (b) Multiple seed models initiate independent refinements of C8. To minimize distortions of the RCT structures, RCT starting models were used as references for Euler angular assignment of reference-free generated 2D class averages in EMAN[2]. Resulting 3D reconstructions exhibited density features similar to those found in the final reconstruction starting from the Gaussian blob (grey). These improved RCT structures were then used as templates for the refinement of single particles (see Fig. S3). Scale bar, 45 Å.

Fig S3. Iterative and parallel refinements of C8 from different starting models using EMAN[2] confirm density features in the reconstruction and show minimal model bias. The C8 reconstruction refined from the Gaussian blob (grey) is compared with a reconstruction refined using one of the models from the random conical tilt method (yellow, as colored in Fig. S2). Scale bar, 45 Å.

Fig S4. Fourier Shell Correlation for the final C8 reconstruction whose refinement was initiated using the Gaussian blob.

Fig S5. Two possible placements for C8β-MACPF as described in Table S1. For each placement, C8β-MACPF is rendered as an isosurface filtered to 25 Å (green). Crystal structures of C8α-MACPF and C8γ are shown as blue and yellow ribbons, respectively.

The C8 reconstruction is highlighted as a grey mesh. N and C termini of C8α-MACPF are cyan spheres, N and C termini of C8β-MACPF are brown spheres. Scale bar, 45Å.

Fig S6.

Domain boundaries of C8 subunits. The SCOP prediction program was used to define domains of the human C8α and C8β sequences. The domains were further truncated to the N and/or C terminal conserved cysteine residues for the N-terminal TSP1, LDLRA, EGF, and C-terminal TSP1 domains of Cα and C8β and the MACPF domain of C8β. The hairpin extension of C8α-MACPF that is covalently linked to C8γ is shown as residues 176-208.

Supplementary Tables

Table S1. Results of fitting and refining atomic models of C8 subunits into a 3D reconstruction of C8 at 24 Å resolution

**Model**[*]	**R_{factor}**[¶]	**CC**[§]
γ-α(MACPF)	43	69
γ-α(MACPF), β(MACPF) [a]	40	84
γ-α(MACPF), β(MACPF) [b]	38	84

[*] The model was generated in a stepwise fashion beginning with manual placement of the C8α-MACPF-C8γ crystal structure, γ-α(MACPF). Its position was refined as a single rigid-body using PHENIX[7]. Two possible placements of a homology model of C8β MACPF, β(MACPF)[a] and β(MACPF)[b], were manually fit in the remaining density and subsequently rigid-body refined. These two placements are related by a 180° rotation interchanging the arms of the "L".

[¶] R_{factor} is the crystallographic R factor minimized during the PHENIX[7] refinement.

[§]CC is the real space correlation coefficient between the EM reconstruction and the map calculated from the model. The value was generated using the BSOFT[5] program suite.

Supplementary References

1. Abraha, A., Morgan, B.P. and Luzio, J.P. (1988) The preparation and characterization of monoclonal antibodies to human complement component C8 and their use in purification of C8 and C8 subunits. *Biochem. J.*, **251**, 285-292.
2. Ludtke SJ, Baldwin PR, Chiu W. (1999) EMAN: semiautomated software for high-resolution single-particle reconstructions. *J. Struct. Biol.*, **128**(1):82-97.
3. Radermacher, M., Wagenknecht, T., Verschoor, A. and Frank, J. (1987) Three-dimensional reconstruction from a single-exposure, random conical tilt series applied to the 50S ribosomal subunit of Escherichia coli. *J. Microsc.*, **146**, 113-136.
4. Frank, J., Radermacher, M., Penczek, P., Zhu, J., Li, Y., Ladjadj, M. and Leith, A. (1996) SPIDER and WEB: processing and visualization of images in 3D electron microscopy and related fields. *J. Struct. Biol.*, **116**, 190-199.
5. Heymann, J.B. (2001) Bsoft: image and molecular processing in electron microscopy. *J. Struct. Biol.*, **133**, 156-169.
6. Penczek, P., Radermacher, M. and Frank, J. (1992) Three-dimensional reconstruction of single particles embedded in ice. *Ultramicroscopy*, **40**, 33-53.
7. Adams, P. D., Afonine, P. V., Bunkoczi, G., Chen, V. B., Davis, I. W., Echols, N., Headd, J. J., Hung, L. W., Kapral, G. J., Grosse-Kunstleve, R. W., McCoy, A. J., Moriarty, N. W., Oeffner, R., Read, R. J., Richardson, D. C., Richardson, J. S., Terwilliger, T. C., and Zwart, P. H. (2010) *Acta Crystallogr. Sect. D: Biol.* **66**, 213-221.

Fig. S1

a

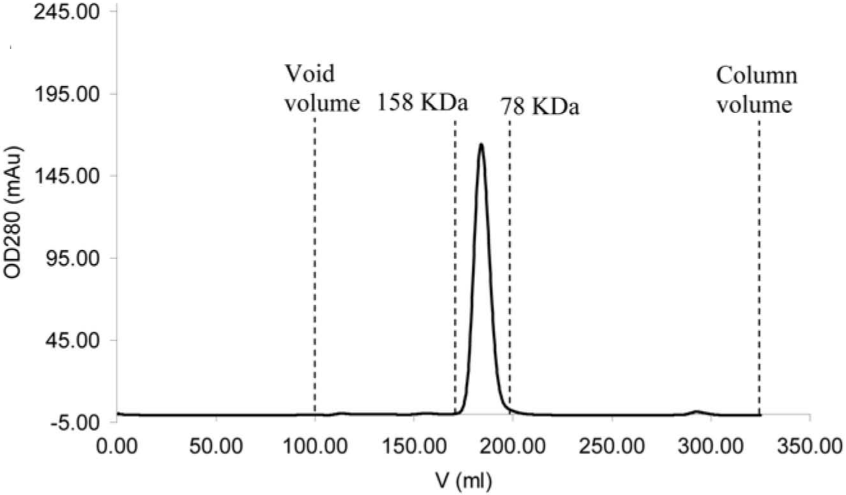

245.00
195.00
145.00
95.00
45.00
-5.00
OD280 (mAu)
Void volume
158 KDa
78 KDa
Column volume
0.00
50.00
100.00
150.00
200.00
250.00
300.00
350.00
V (ml)

b

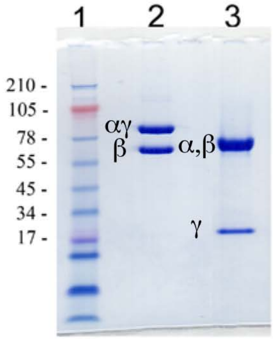

1
2
3
210 -
105 -
78 -
55 -
45 -
34 -
17 -
αγ
β
α,β
γ

Fig. S2

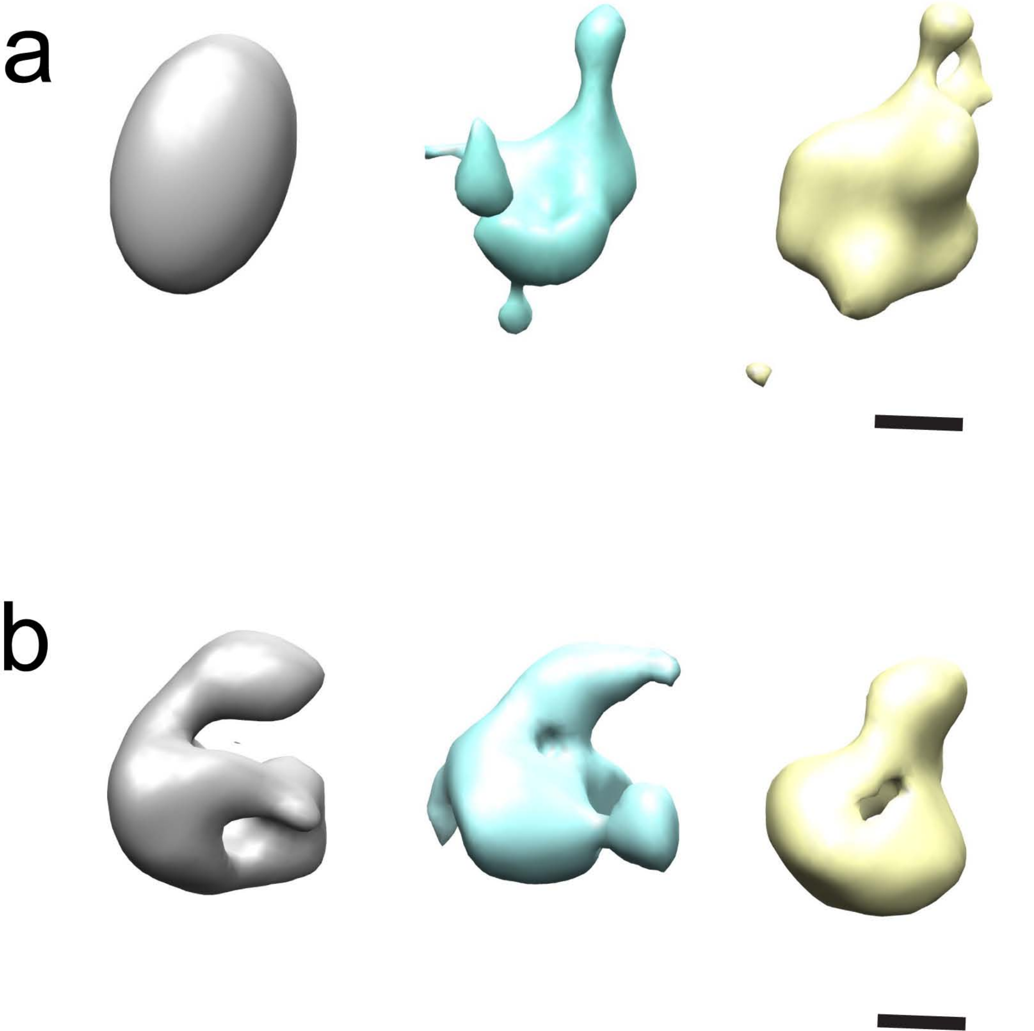

Fig. S3

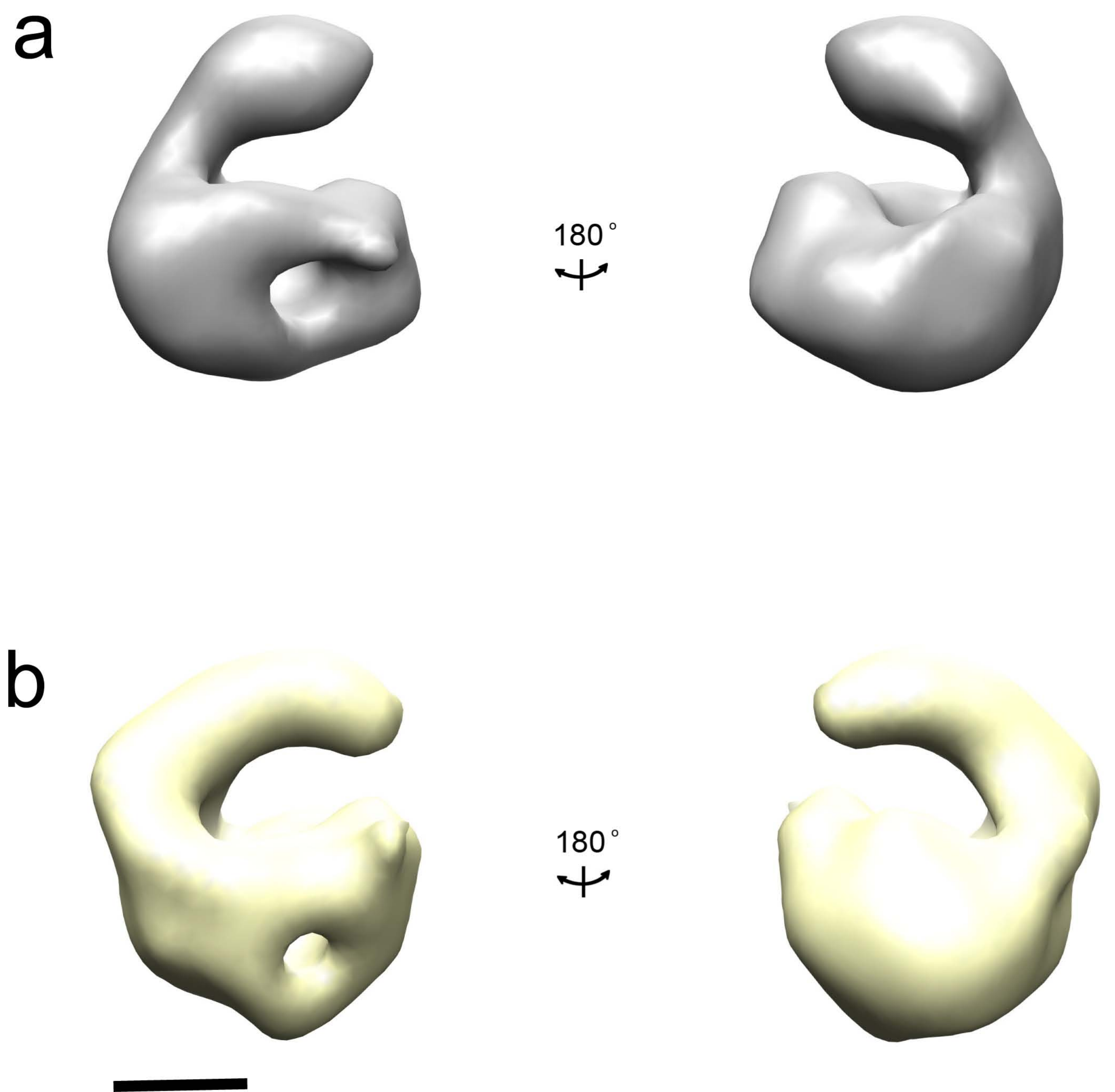

Fig. S4

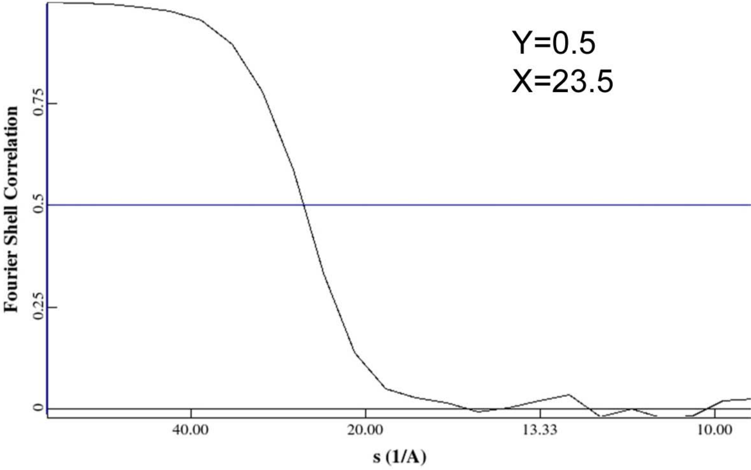
Y=0.5
X=23.5
Fourier Shell Correlation
0.75
0.5
0.25
0
40.00
20.00
13.33
10.00
s (1/A)

Fig. S5

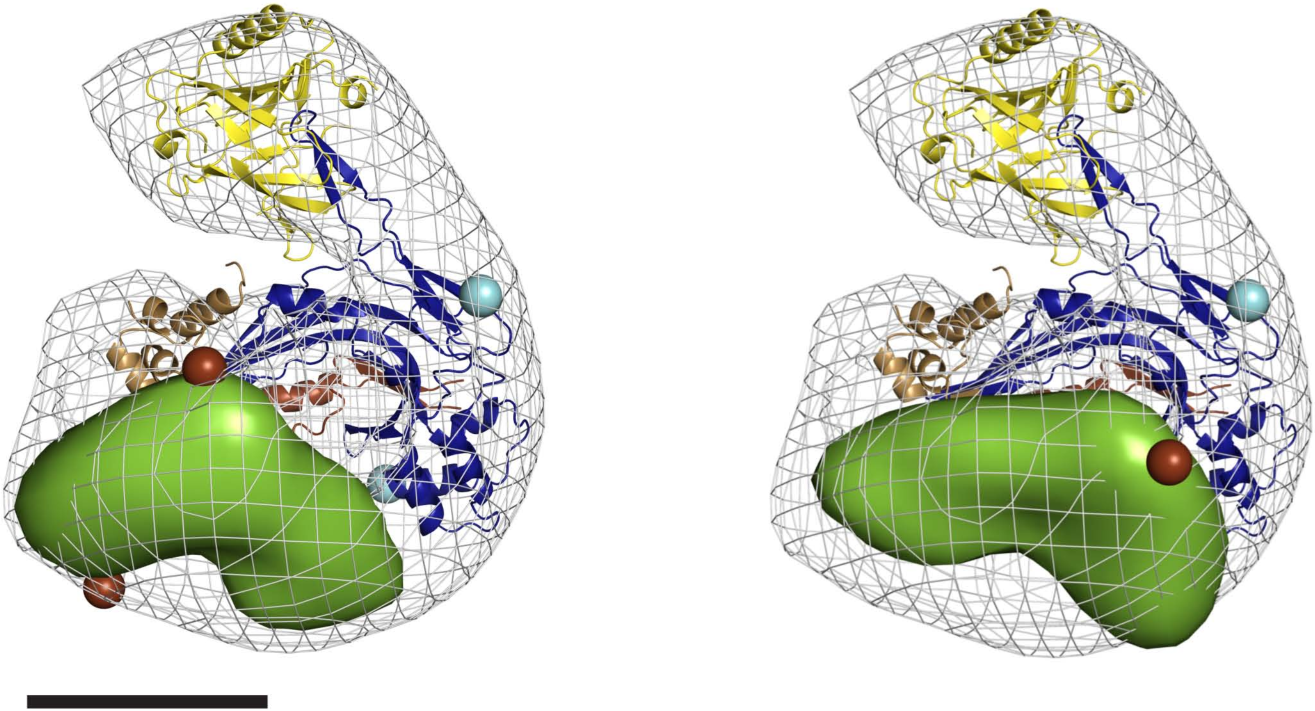

Fig. S6

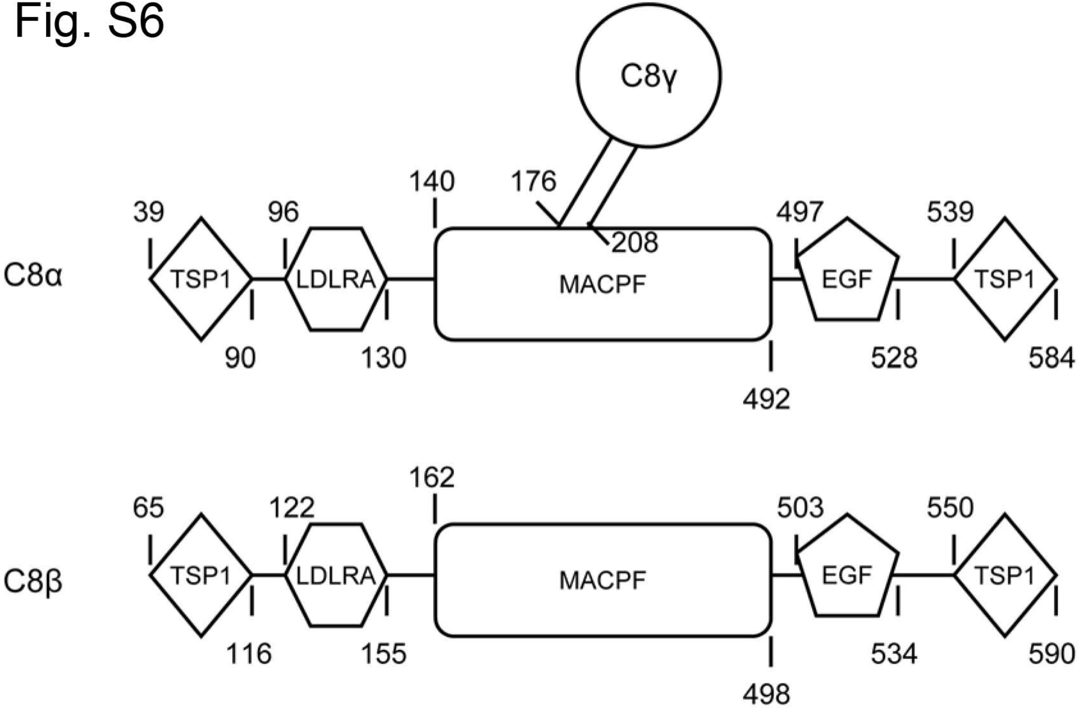
C8γ
176
208
140
39
96
497
539
C8α
TSP1
LDLRA
MACPF
EGF
TSP1
90
130
528
584
492
162
65
122
503
550
C8β
TSP1
LDLRA
MACPF
EGF
TSP1
116
155
534
590
498
